# Neutrophil-Mediated Phagocytic Host Defense Defect in Myeloid Cftr-Inactivated Mice

**DOI:** 10.1371/journal.pone.0106813

**Published:** 2014-09-03

**Authors:** Hang Pong Ng, Yun Zhou, Kejing Song, Craig A. Hodges, Mitchell L. Drumm, Guoshun Wang

**Affiliations:** 1 Department of Microbiology and Immunology, Louisiana State University Health Sciences Center, New Orleans, Louisiana, United States of America; 2 Department of Pediatrics, Case Western Reserve University School of Medicine, Cleveland, Ohio, United States of America; 3 Department of Genetics and Genome Sciences, Case Western Reserve University School of Medicine, Cleveland, Ohio, United States of America; 4 Department of Genetics, Louisiana State University Health Sciences Center, New Orleans, Louisiana, United States of America; 5 Department of Medicine, Louisiana State University Health Sciences Center, New Orleans, Louisiana, United States of America; University of Bern, Switzerland

## Abstract

Cystic fibrosis (CF) is a common and deadly inherited disease, caused by mutations in the CFTR gene that encodes a cAMP-activated chloride channel. One outstanding manifestation of the disease is the persistent bacterial infection and inflammation in the lung, which claims over 90% of CF mortality. It has been debated whether neutrophil-mediated phagocytic innate immunity has any intrinsic defect that contributes to the host lung defense failure. Here we compared phagosomal CFTR targeting, hypochlorous acid (HOCl) production, and microbial killing of the neutrophils from myeloid Cftr-inactivated (Myeloid-Cftr−/−) mice and the non-inactivated control (Cftr^fl10^) mice. We found that the mutant CFTR that lacked Exon-10 failed to target to the neutrophil phagosomes. This dysfunction resulted in impaired intraphagosomal HOCl production and neutrophil microbial killing. *In vivo* lung infection with a lethal dose of *Pseudomonas aeruginosa* caused significantly higher mortality in the myeloid CF mice than in the controls. The myeloid-Cftr−/− lungs were deficient in bacterial clearance, and had sustained neutrophilic inflammation and stalled transition from early to late immunity. These manifestations recapitulated the symptoms of human CF lungs. The data altogether suggest that myeloid CFTR expression is critical to normal host lung defense. CFTR dysfunction in neutrophils compromises the phagocytic innate immunity, which may predispose CF lungs to infection.

## Introduction

Cystic Fibrosis (CF) is the most common genetic disease in Caucasians with an occurrence of 1/∼3000 live births [1], [2]. It is caused by mutations in the CF transmembrane conductance regulator (CFTR) gene which encodes for a cAMP-activated chloride channel. Even though CF affects multiple organs and systems, the most severe and life-threatening pathology occurs in the lung, which claims over 90% of CF mortality. Clinical manifestations include persistent bacterial infection and inflammation, prominent neutrophil infiltration, and purulent small airway obstruction. These symptoms imply that CF lungs have an impaired host defense. However, the true link between the chloride channel defect and the host defense failure in CF lungs has not been fully established.

Host lung defense reflects the combined activities of lung resident cells, such as pulmonary epithelial cells and tissue macrophages, and lung-recruited immune cells, most notably neutrophils and monocytes. The lungs of CF patients are remarkably neutrophilic and inflamed [3], indicating that a successful inflammatory response can be mounted by the host. In spite of the robust host response, CF lungs cannot resolve infections. Thus, it is the quality, not the quantity, of the host defense that falls short in CF. Many aspects of functional disturbance or behavior aberrance in CF neutrophils have been previously recognized [4], [5], [6], [7], [8], including suboptimal activation [9], cleavage of CXCR1 [10], hyper-sensitivity to LPS stimulation [11], deviant production of reactive oxygen species [12], genome-wide gene expression perturbation [13], alteration in inflammatory signaling [14], hyper-production of IL-8 [15], [16], delayed apoptosis [17], abnormal extracellular trap formation [18], hyper-oxidation of glutathione [19], and lately abnormal granule release [20]. Neutrophils are professional phagocytes constituting ∼60–70% of the circulating leukocytes in humans. Their major function is to control and eradicate infections, especially extracellular bacterial infection. One of the pivotal microbial killing mechanisms in neutrophils is to produce microbicidal oxidants [21], [22], [23], such as O_2_^−^, H_2_O_2_ and hypochlorous acid (HOCl). Among them, HOCl, the chlorine bleach, has the greatest potency due to its reactivity with almost all macromolecules from lipids to proteins to nucleic acids [24], [25]. Notably, neutrophils use chloride to synthesize HOCl in their phagosomes [22], [26], [27]. This biosynthesis is catalyzed by myeloperoxidase (MPO), an enzyme exclusively expressed in neutrophils [28]. Because chloride is a charged ion, it cannot permeate lipid membranes unless transported through channels or transporters. An early study by Yoshimura and colleagues indicates that CFTR mRNA is transcribed in mature human neutrophils [29]. We have demonstrated that the CFTR channel protein is expressed in human neutrophils [30] and specifically targets to the phagosomes [31]. Further studies have proved that CFTR defect in the neutrophils from the patients with CF impairs the intraphagosomal HOCl production and microbial killing by the phagocyte [30], [32], [33]. However, these findings from CF patients have not been validated in any CF animal models. In the present study, we have used the myeloid tissue-specific Cftr−/− mice to interrogate CFTR expression and function in phagocytic host defense. *In vitro* and *in vivo* experiments have corroborated the critical role of myeloid CFTR in phagocyte-mediated lung host defense. Impairment of such a mechanism alone is sufficient to damage the lung’s ability to defend against Pseudomonas infection.

## Materials and Methods

### Ethics statement

This research involves use of animals. The study was carried out in strict accordance with the recommendations in the Guide for the Care and Use of Laboratory Animals of the National Institutes of Health. All procedures involving animal use were reviewed and approved by the Louisiana State University Health Sciences Center Animal Care and Use Committee (IACUC #2836). For anesthesia, isoflurane inhalation was used prior to lung bacterial intubation. Carbon dioxide suffocation was used to euthanize animals.

### Chemicals and solutions

All common chemicals were obtained from Sigma-Aldrich. The physiological chloride Ringer’s buffer was composed of 122 mM NaCl, 1.2 mM MgCl_2_, 1.2 mM CaCl_2_, 2.4 mM K_2_HPO_4_, 0.6 mM KH_2_PO_4_, 20 mM HEPES (pH 7.3), and 10 mM dextrose. The gluconate chloride-free Ringer’s buffer was made by substitution of the chloride salts with equal molar concentration of gluconate salts except 4 mM of calcium gluconate was used to compensate for the mild calcium chelating effect of gluconate. The KCl relaxation buffer contained 50 mM KCl, 3 mM NaCl, 3.5 mM MgCl_2_, 0.5 mM EGTA, 1 mM ATP, 20 mM PIPES (pH 7.0), 1 mM PMSF and Sigma protease inhibitor cocktail.

### Mice

Creation of the *Cftr* Exon 10-floxed mice, referred to here as *Cftr^fl10^*, was published [34]. As documented previously [35], the myeloid CFTR-Exon-10-deleted (Myeloid-Cftr−/−) mice were produced by breeding the *Cftr^fl10^* mice with the LysMCre transgenic mice [36]. After repeated backcross and screen, the *Cftr^fl10^* homozygous mice carrying the LysMCre transgene were selected by PCR genotyping for experiments.

### Lung bacterial infection and animal survival assay

*Pseudomonas aeruginosa* bacteria (PsA, clinical isolate) were embedded in agarose beads for chronic lung infection. Preparation of the PsA-laden beads was modified from the previous description [37], [38]. Briefly, overnight culture of PsA in tryptic soy broth (TSB) was washed and resuspended in fresh TSB to give a final concentration of ∼1×10^8^ cfu/ml. Low-melting temperature agarose was melted in the gluconate chloride-free Ringer’s buffer to make a final concentration of 2%, which was then equilibrated to 45°C and mixed well with PsA. Then, the mixture was poured into 45°C pre-warmed heavy mineral oil (Sigma-Aldrich). The mixture was cooled on ice gradually with agitation. The agarose slush was washed with the gluconate chloride-free Ringer’s buffer, and homogenized (3 strokes, 10 seconds per stroke at the full speed) with a polytron homogenizer (Omni International Inc., Warrenton, VA, USA). Serial dilutions were made and plated in duplicates on LB plates for colony-forming unit determination. After obtaining the bacterial titer, the PsA-laden beads were non-invasively instilled into mouse lungs under anesthesia [39]. For lethal challenge, a total of 5×10^6^ cfu of the agarose-embedded PsA in 50 µl of suspension was intubated. The bacterium-challenged mice were monitored twice a day for 7 days. Behavior change and mortality were recorded. If animals showed severe pain or stress symptoms such as weight loss, dehydration, incontinence, eyes sunken, lids closed, wasting of muscles on back, sunken or distended abdomen, decreased vibrissae movement, unresponsive, ataxia, circling, hypothermia, analgesics (Buprenorphine; 0.1 mg/kg, SQ) was administered. Dying animals with weight loss up to 20% were considered mortality and euthanized by CO_2_ asphyxiation. All survived animals were euthanized a week after bacterial challenge.

### Bronchoalveolar lavage (BAL) and cytodifferential determination

The PsA-infected mice, euthanized by CO_2_ inhalation, were dissected to expose their tracheas. Blunt needles (23G), were inserted into the tracheas to cannulate the lungs, and secured with a silk suture. Ice-cold PBS lavage solution (1 ml) containing the protease inhibitor cocktail (Roche) was used to flush each lung and held for 1 minute before aspirating out. Then, additional 0.5 ml lavage solution was used for a second lung wash. Typically, a total of ∼1.2 ml of BAL fluid per mouse was recoverable by the procedure. BAL cell differentials were obtained by cytospin, Giemsa staining and microscopic assessment.

### Peripheral blood neutrophil purification

Peripheral blood neutrophils were isolated following the method modified from the previous description [40]. Briefly, mouse blood (350±50 µl per animal) was collected by retro-orbital bleeding into PBS-EDTA solution (PBS without Ca^2+^ and Mg^2+^, pH 7.2, 15 mM EDTA, and 1% bovine serum albumin). After centrifugation (400×*g*) for 10 min at 4°C, cells were resuspended in 0.2 ml PBS-EDTA solution. Six to ten mouse blood samples were pooled and laid onto a three-layer Percoll gradient (78%, 69%, and 52% Percoll PLUS) (GE Healthcare Biosciences, PA, USA). After centrifugation for 30 min at 1500×*g* at room temperature without braking, the neutrophil layer at the 69% and 78% interface was collected into a 1% BSA-coated tube. After one wash with 2 ml PBS-EDTA, the red cells were lysed by hypotonic shock. After a final wash with 2 ml PBS-EDTA, the neutrophils were resuspended in the chloride-free gluconate Ringer’s buffer to deplete chloride for 1 hour on ice for use.

### Peritoneal cavity neutrophil and monocyte isolation

In the case of isolating peritoneal neutrophils and monocytes, we followed the published protocol [41]. Briefly, 9% casein solution was intraperitoneally injected 12 hours and 3 hours prior to peritoneal exudate harvest. Then, the collected peritoneal neutrophils and monocytes were further purified by Percoll-gradient centrifugation as described above. Giemsa staining and microscopic examination confirmed that this protocol resulted in over 90% purity.

### Neutrophil CFTR immunostaining

Percoll-gradient purified neutrophils from peripheral blood were fixed with 4% paraformaldehyde for 30 minutes at room temperature, and permeabilized with 0.5% Triton X-100/PBS for 30 minutes. The cells were then blocked with Blocking buffer (5% normal goat serum in 0.1% Triton X-100/PBS) for 1 hour, and incubated with the CFTR-specific antibody [42] (mAb #660, CFTR Antibody Distribution Program, UNC/CFF Therapeutics, Inc.) or IgG2b isotype control antibody in Blocking buffer for 2 hours at room temperature. Next, the cells were washed with PBS and subsequently incubated with the PE-conjugated goat–anti-mouse IgG secondary antibody (Invitrogen, Camarillo, CA, USA) for 45 minutes. Expression levels were analyzed by flow cytometry.

### Phagosomal CFTR immunostaining

Latex beads (3 µm, Sigma-Aldrich) were coated with BSA and anti-BSA antibody and serum-opsonized similarly as previously described [31]. Purified mouse peripheral blood neutrophils were mixed with the opsonized beads and incubated for 30 min at 37°C with shaking, followed by washes with PBS by low speed centrifugation at 100×*g* to remove the non-phagocytosed beads. Then the cells were incubated in protease inhibitor cocktail on ice for 10 min to inactivate serine proteases, then rinsed with 1 ml of the KCl relaxation buffer with 1 mM ATP. Finally, 0.2 ml of the same relaxation buffer containing protease inhibitor cocktail (Roche) was added. The cells were immediately subjected to homogenization by going through 27G needle for 5 times. The lysate was collected and centrifuged (400×*g*) at 4°C for 5 min to remove intact cells and nuclei. The phagosome-containing supernatant was recovered for immunostaining with the primary CFTR antibody or the isotype control, in the presence of 5% normal goat serum, followed by staining with the PE-conjugated goat anti-mouse IgG. The samples were post-fixed with the same volume of 2% paraformaldehyde/PBS and analyzed immediately by flow cytometry. The instrument was electronically gated on the clearly distinguishable 3-µm bead/phagosome population. Our preliminary study proved that ∼70–80% of the gated population was positive for Lysosomal-associated membrane protein-1, indicating that the majority of the gated events were mature phagosomes.

### Intraphagosomal hypochlorous acid measurement

HOCl production in neutrophils was examined using the HOCl-specific fluorescent probe R19-S [43], [44]. R19-S in its natural state does not fluoresce. However, HOCl oxidation renders it fluorescent [43]. Neutrophils were fed serum-opsonized PsA at a ratio of 1∶20 in chloride-free Ringer’s buffer for 15 min. After low speed centrifugation, the cells with phagocytosed bacteria were resuspended in the Ringer’s buffer with or without chloride for 20 min. Then, R19-S (10 µM) was added to the cell suspension for additional 15 minutes. Fluorescence intensity of R19-S was analyzed by flow cytometry.

### Neutrophil-mediated bacterial killing

Bacterial killing assay was performed using our published protocol with modifications [33]. Neutrophils purified from mouse peripheral blood were resuspended in the chloride-free Ringer’s buffer on ice for 1 hour to deplete the intracellular free chloride. The cells (5×10^5^) were plated in a well of a 48-well plate for 20 min at 37°C. After cell adhesion, the supernatant was aspirated and the serum-opsonized PsA bacteria in the phagocytosis buffer (chloride-free Ringer’s buffer supplemented with 10% chloride-depleted mouse normal serum) were added for phagocytosis for 15 min at 37°C. Next, the non-phagocytosed bacteria were washed away with 0.5 ml ice-cold chloride-free Ringer’s buffer three times. Then the cells were incubated in either no-chloride (0 mM) or NaCl (122 mM) Ringer’s buffer for additional 40 minutes at 37°C. The number of viable bacteria after killing was determined by LB agar plating as described before [33].

### Determination of lung bacterial clearance

Mice were intubated intratracheally with agarose-embedded PsA at the sub-lethal dose (1×10^6^ cfu). The animals were then sacrificed at Day 0 and Day 3. The lungs were lavaged with ice-chilled PBS (1 ml) and the bacterial number in each BAL fluid was determined by LB agar plating. Then, the total bacterial number in each BAL fluid was back calculated.

### Statistics

Data were statistically analyzed by Student’s t-test for differences between two comparing groups. The animal survival data were compared by Log-Rank test. Results were expressed as mean ± SD. Differences with P-values smaller than 0.05 were considered statistically significant.

## Results

### Specific deletion of CFTR Exon-10 in myeloid lineages in mice

**Figure 1:**
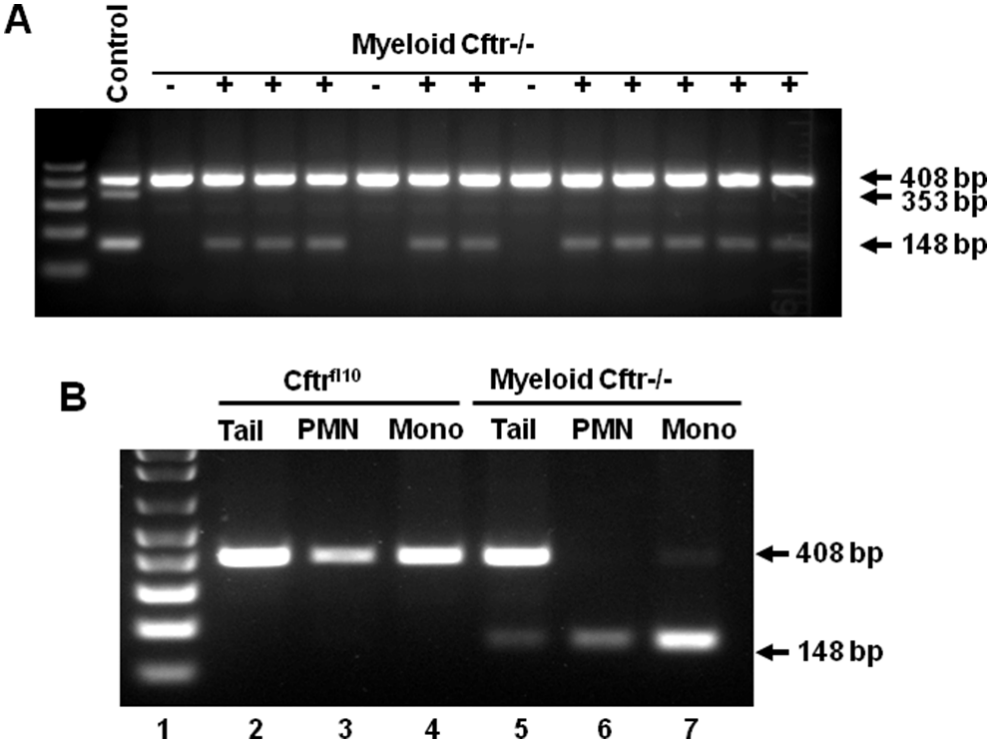
Myeloid CFTR-inactivation in mice. Myeloid tissue-specific CFTR-Exon-10 deletion mice (Myeloid-Cftr−/−) were produced by breeding the Cftr^fl10^ mice with the LysMCre transgenic mice. The Cftr^fl10^ mice had a double-allele modification of CFTR Exon-10 which was flanked by loxP sites, while the LysMCre mice had myeloid Cre-recombinase expression under the control of the LysM gene promoter. Myeloid-Cftr−/− mice were screened by PCR-genotyping using the specific primers to amply CFTR Exon-10. **A**) DNA gel of PCR products from the tail segments of two litters of pups. The 408-bp PCR amplicon indicates an intact and floxed CFTR Exon-10. In contrast, the 148-bp amplicon shows a deleted CFTR-Exon-10. The control lane shows the PCR products of a mixture of DNAs from the tails of wild-type, Cftr^fl10^ and Myeloid-Cftr−/− mice. The 353-bp amplicon represents the non-floxed, wild-type CFTR Exon-10. **B**) Confirmation of myeloid CFTR Exon-10 deletion. Purified neutrophils (PMN) and monocytes (Mono), and the corresponding tail segment (Tail) were used for PCR-genotyping. Both neutrophils and monocytes from the Myeloid-Cftr−/− mouse have the 148-bp product, indicating Exon-10 deletion, whereas the phagocytes from the control Cftr^fl10^ mouse show the 408-bp product. Contrarily, tails from both types of mice display the double bands due to the mixture of multiple cell types.

Myeloid tissue-specific Cftr−/− mice have been established and characterized in the previous report [35]. These mice express functional CFTR in all tissues except myeloid lineages in which CFTR Exon-10 is specifically deleted. Notably, their intestinal and pulmonary epithelia have normal CFTR function [35]. Thus, this model would allow us to define the potential role of myeloid CFTR in phagocytic host defense in the lungs without interference of other cell types. To ensure that all animals used for the current study had the designed tissue-specific CFTR Exon-10 deletion, we PCR-genotyped all the pups from the myeloid Cftr−/− breeding colony. Shown in **Figure 1A** is a representative DNA gel of the PCR products from the tail segments of different animals, displaying that 10 of the 13 tested mice had the Exon-10 deletion, which gave rise to the small PCR-amplicon (148 bp). The large PCR-amplicon (408 bp) indicates an intact and floxed Exon-10. Because the tail segments contained epithelial, fibroblast and blood cells, a mixture of Exon-10 deletion and non-deletion was detected in each PCR reaction from the Cre-positive mice. To confirm the deletion was myeloid lineage-specific, we isolated and purified the peritoneal neutrophils and monocytes from both non-deletion Cftr^fl10^ control and Myeloid-Cftr−/− mice. PCR-genotyping of the phagocytes and the corresponding tail segments was performed and compared. As displayed in **Figure 1B**, all the Cftr^fl10^ samples exhibited an intact floxed CFTR Exon-10 with the large amplicon (408 bp). In contrast, the myeloid Cftr−/− samples demonstrated the desired Exon-10 deletion with the small PCR-product (148 bp) in neutrophils and monocytes.

### Myeloid CFTR Exon-10 deletion impairs CFTR presentation to neutrophil phagosomes

**Figure 2:**
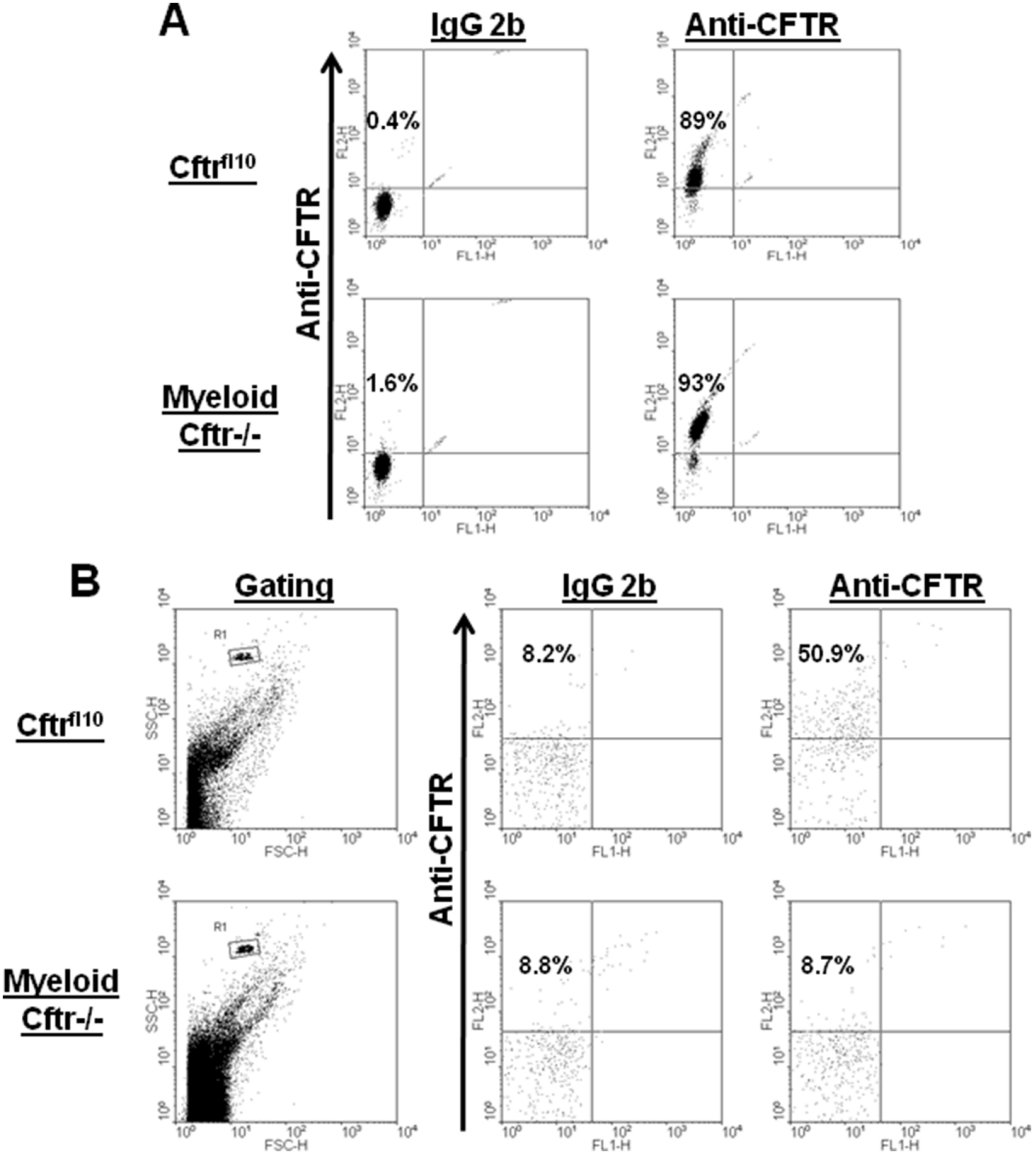
Flow cytometric analysis of CFTR expression in neutrophils and phagosomes. **A**) CFTR staining in neutrophils. Purified peripheral-blood neutrophils were immunostained for intracellular CFTR using the monoclonal CFTR antibody (mAb #660). Representative dot plots show that the neutrophils from Myeloid-Cftr−/− mice express CFTR (93%), and the cells from the control Cftr^fl10^ mice are 89% positive for CFTR. In contrast, the isotype control antibody shows little background staining. **B**) CFTR staining in phagosomes. Latex beads (3 µm) were phagocytosed by neutrophils from the control Cftr^fl10^ mice or the Myeloid-Cftr−/− mice. Phagosomes released by cell homogenization were immunostained with the same anti-CFTR antibody. The phagosomal population was gated by flow cytometry. Only the phagosomes from the control Cftr^fl10^ neutrophils show significant staining for CFTR.

To determine whether the mutant CFTR without Exon-10 had any protein expression in myeloid cell lineages, we immunostained the peripheral blood neutrophils from Cftr^fl10^ control and Myeloid-Cftr−/− mice with the CFTR-specific antibody described in Materials and Methods. Flow cytometric analysis (**Fig. 2A**) demonstrated that there was no significant difference in the levels of overall CFTR expression in the phagocytes from the two types of mice. Next, we decided to compare CFTR targeting to the phagosomes of neutrophils. Peripheral blood neutrophils were fed serum-opsonized latex beads, homogenized to release their phagosomes which were then immunostained with the CFTR-specific antibody. As displayed (**Fig. 2B**), flow cytometry analysis revealed that the phagosomes from Myeloid-Cftr−/− neutrophils had little CFTR staining, while about half of the Cftr^fl10^ phagosomes were stained positive. These data suggest that the deletion of CFTR Exon-10 in myeloid lineages impairs CFTR presentation to the phagosomes, but not its overall CFTR protein expression in the cells.

### Myeloid CFTR-Exon-10 deletion impairs intraphagosomal HOCl production in neutrophils

**Figure 3:**
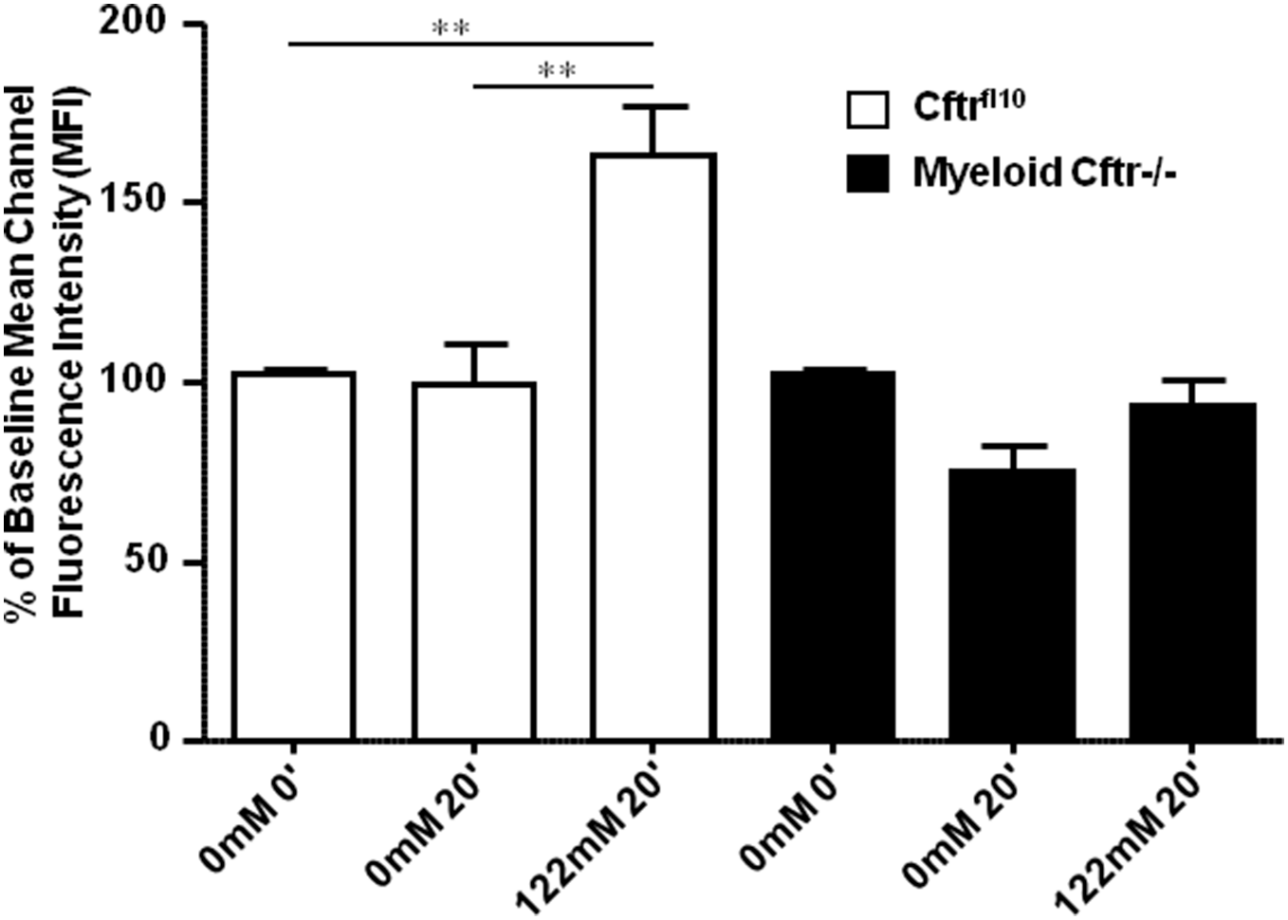
Impairment of phagosomal HOCl production in neutrophils from Myeloid Cftr−/− mice. Neutrophil production of HOCl was assessed using the fluorescent HOCl probe R19-S. Phagocytosis was performed by mixing neutrophils with opsonized PsA in the chloride-free buffer (0 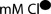) for 15 min, and the cells were further incubated in the buffer containing either 0 mM or 122 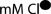 for 20 min. Then, R19-S was added to incubate for an additional 15 min. Fluorescence of R19-S produced by HOCl oxidation was analyzed by flow cytometry. Mean fluorescence intensity (MFI) of each sample was obtained and compared to their 0-min time point. Statistical difference was judged by Student’s t-test. Double asterisks indicate significant differences (p<0.01, n = 4).

Neutrophils need chloride to synthesize HOCl in their phagosomes. Since CFTR is a chloride channel located to the phagosomal membrane, we predicted that its dysfunction might affect phagosomal chloride attainment and HOCl production, as found in human CF neutrophils [30]. To prove this prediction, we measured phagosomal oxidation of R19-S, a fluorescent probe specific for HOCl. Percoll-gradient purified peripheral blood neutrophils from both Cftr^fl10^ control and Myeloid Cftr−/− mice were kept on ice in the chloride-free Ringer’s buffer for 1 hour in order to deplete the intracellular free chloride. After phagocytosis of serum-opsonized PsA, the cells were resuspended in either chloride-free (0 mM) or physiological chloride Ringer’s buffer (122 mM) for 20 minutes, followed by R19-S application for additional 15 minutes. This protocol was designed to detect HOCl generated from the chloride acquired from the extracellular buffer after phagocytosis. R19-S fluorescence from HOCl oxidation was quantitatively analyzed by flow cytometry. **Figure 3** shows that the mean fluorescence intensity (MFI) of the R19-S-positive neutrophils was significantly increased only in the case of control Cftr^fl10^ cells in 122 mM chloride Ringer’s buffer. In contrast, the neutrophils with the CFTR-Exon-10 deletion did not show any surge of HOCl production even with ample chloride supply in the 122 mM chloride medium. These data suggest a deficit in HOCl production by Myeloid-Cftr−/− neutrophils.

### Myeloid CFTR Exon-10 deletion impairs neutrophil microbial killing

**Figure 4:**
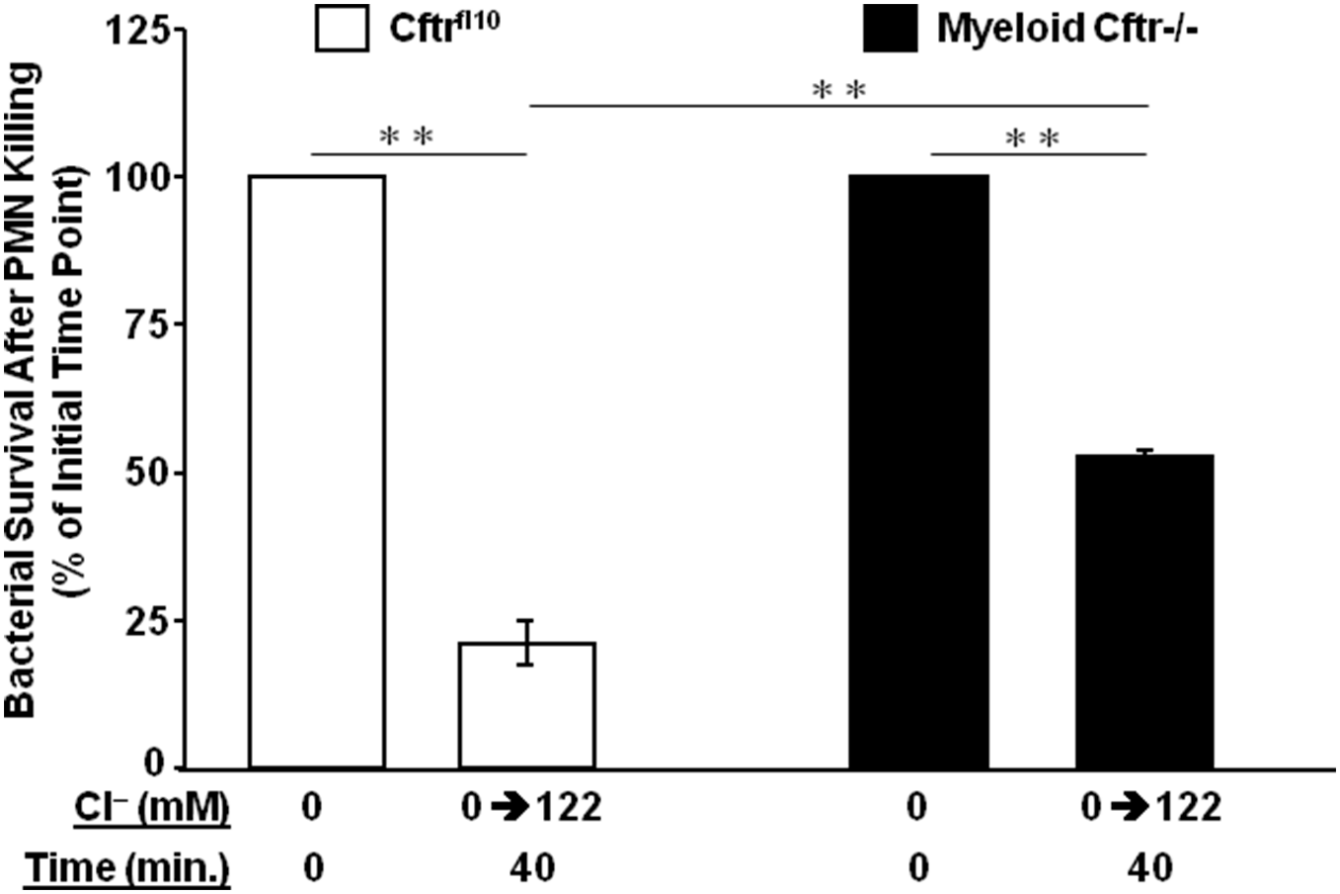
Bacterial killing deficiency of neutrophils from Myeloid-Cftr−/− mice. Neutrophils, isolated from either Cftr^fl10^ mice or Myeloid-Cftr−/− mice, were incubated with serum-opsonized PsA at a ratio of 1∶20 in 0-mM chloride buffer. After washing away the non-phagocytosed bacteria, the cells were further incubated in the buffer containing 122 mM chloride for 40 minutes. After killing, viable PsA was released by lysing the neutrophils with 0.05% Saponion solution and cultured on LB plates for colony counting. Bacterial survival rate was obtained by comparing the colony forming units before and after neutrophil killing. Statistical differences were judged by Student’s t-test. Double asterisks indicate significant differences (p<0.01, N = 3).

Phagosomal HOCl production in neutrophils plays a key role in neutrophil-mediated microbial killing [22], [26], [27], [45], [46], [47], [48]. Since the Myeloid-Cftr−/− neutrophils had an impaired HOCl production, we then examined the possible outcome of such a defect with regard to bacterial killing. Serum-opsonized PsA bacteria were phagocytosed in chloride-free Ringer’s buffer by neutrophils isolated from either Cftr^fl10^ control or Myeloid-Cftr−/− mice at a ratio of 1∶20 (PMN:PsA). After removal of the non-phagocytosed bacteria by repeated washing with the chloride-free Ringer’s buffer, further incubation was carried out in the physiological chloride Ringer’s buffer. After 40-min killing, viable PsA bacteria were recovered and cultured overnight on LB plates for colony counting. **Figure 4** shows the bacterial survival rates relative to their initial time points (0 min after killing). Myeloid-Cftr−/− neutrophils had approximately twice as much PsA survival as that of the Cftr^fl10^ cells. Such a killing difference was not due to variation of bacterial uptake, because the numbers of bacteria phagocytosed by the two types of neutrophils, measured at the initial time point of killing, were comparable by Student’s t-test (p = 0.41, N = 3). Thus, myeloid CFTR inactivation undermines the neutrophil microbicidal capacity.

### Myeloid CF Mice have impaired lung host defense

**Figure 5:**
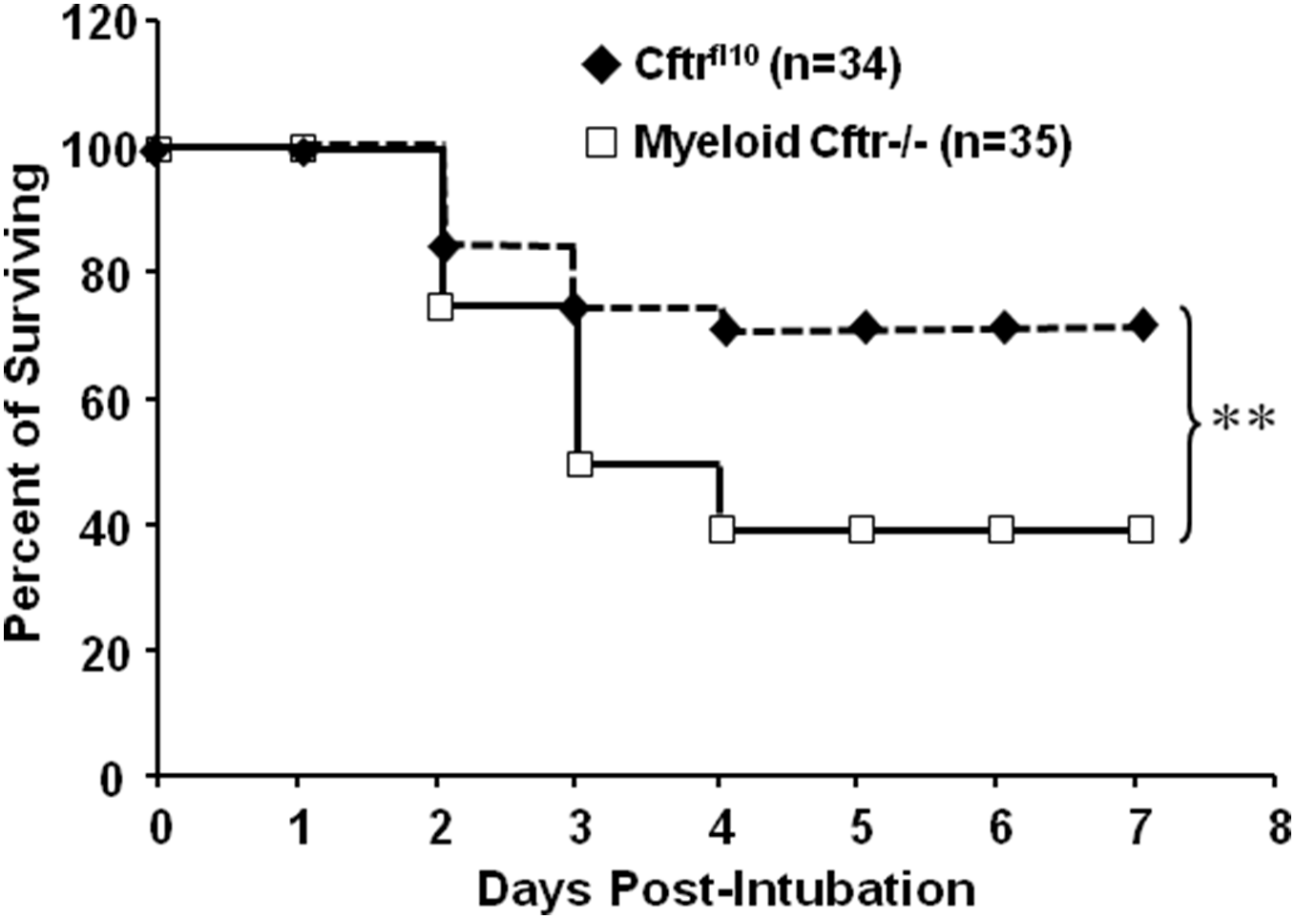
Lung host defense defect in Myeloid-Cftr−/− mice. Myeloid-Cftr−/− mice and Cftr^fl10^ mice were infected with the agarose-embedded PsA at the lethal dose of (5×10^6^ cfu) by lung intubation. Casualties were recorded and survival curves traced. The log-rank test was performed to check statistical difference in survival between the Cftr^fl10^ mice (n = 34) and the myeloid-Cftr−/− mice (*n* = 35). Double asterisks mean significant difference (p<0.01).

To determine if the myeloid CFTR dysfunction alone significantly affects the lung host defense, we intratracheally inoculated Myeloid-Cftr−/− mice and Cftr^fl10^ control mice with the lethal dose of the agarose-embedded PsA (5×10^6^ cfu). As shown in the Kaplan-Meier survival curve (**Fig. 5**), casualties largely occurred over the period of Days 2 to 4 post inoculation. Under the applied level of challenge, Myeloid-Cftr−/− mice had a survival rate of ∼40%, whereas the non-deletion control mice had a survival rate of ∼70%, which was significantly higher. This result concurs with the published data [35], confirming the critical role of CFTR in myeloid cell-mediated lung host defense.

### Myeloid CF mice are deficient in lung bacterial clearance

**Figure 6:**
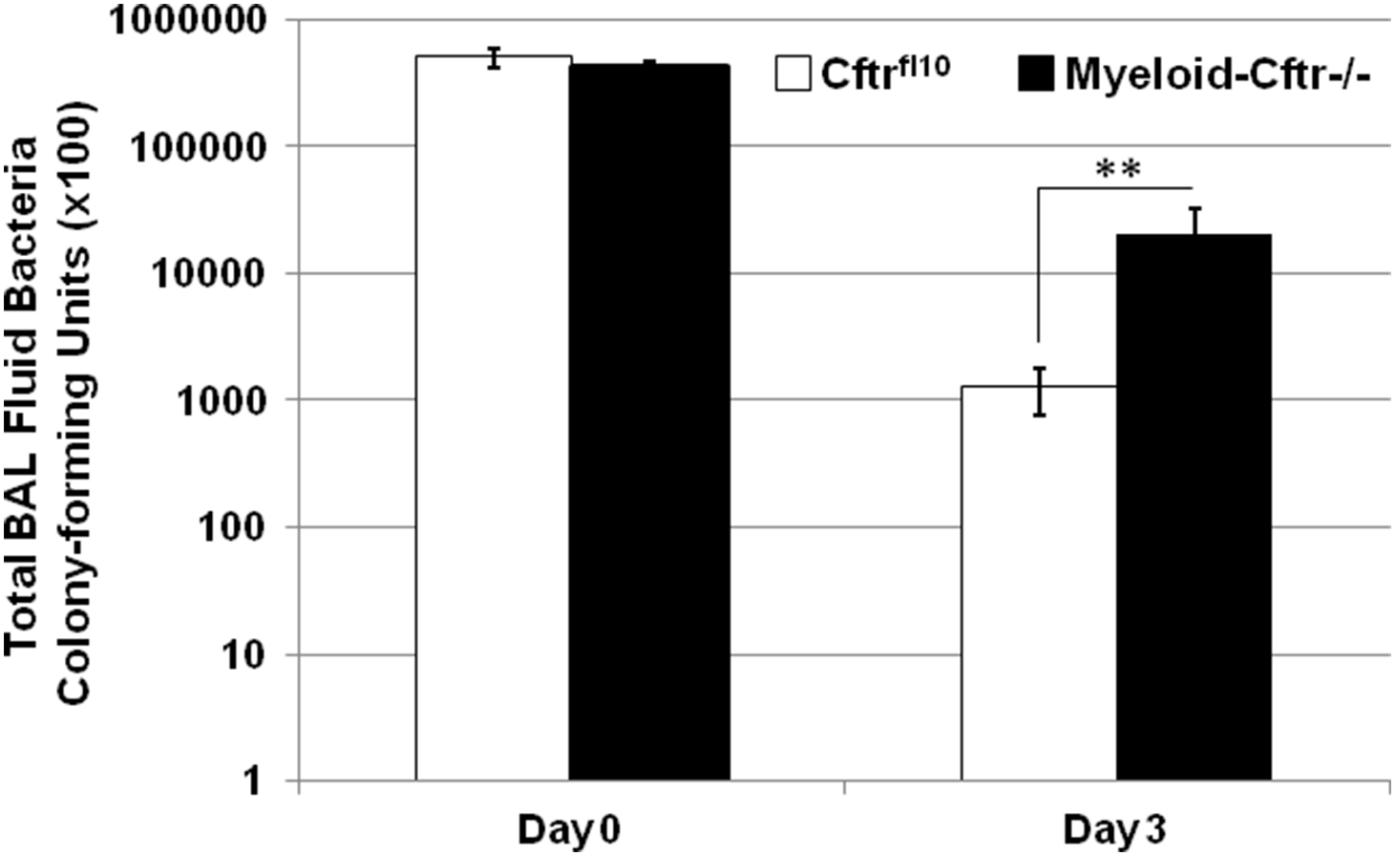
Deficiency in lung bacterial clearance in Myeloid-Cftr−/− mice. Myeloid-Cftr−/− mice and Cftr^fl10^ mice were infected with the agarose-embedded PsA at the sub-lethal dose of (1×10^6^ cfu) by lung intubation. BAL fluids from the animals were collected at Days 0 and 3 after inoculation, and cultured for bacterial quantitation. There was no statistical difference in the total bacterial numbers of the BAL fluids from both types of mice at Day 0 (p = 0.13, n = 4). However, the total BAL bacterial numbers from Myeloid-Cftr−/− mice were significantly higher than those from Cftr^fl10^ mice at day 3 post-infection by Student’s t-test (p<0.01, n = 5).

It is known that the myeloid CF mice have normal CFTR function in airway epithelia [35]. We asked whether the myeloid CFTR inactivation alone cripples the capacity of lung bacterial clearance. Myeloid Cftr−/− mice and Cftr^fl10^ control mice were intubated intratracheally with agarose-embedded PsA at the sub-lethal dose (1×10^6^ cfu). Animals were sacrificed at Day 0 and Day 3. The lungs were lavaged and the bacterial load in each BAL fluid was determined by LB agar plating. As shown in **Fig. 6**, bacterial numbers in the BAL fluids from both Cftr^fl10^ control and Myeloid-Cftr−/− mice were comparable at Day 0 post-inoculation. However, at Day 3 post-inoculation the bacterial numbers of the BAL fluids from the myeloid CF mice were ∼10-fold higher than those from the control mice, suggesting that the myeloid CF mice are deficient in lung bacterial clearance.

### Sustained neutrophilic inflammation and stalled transition from early to late immunity in the lungs of myeloid CF mice

**Figure 7:**
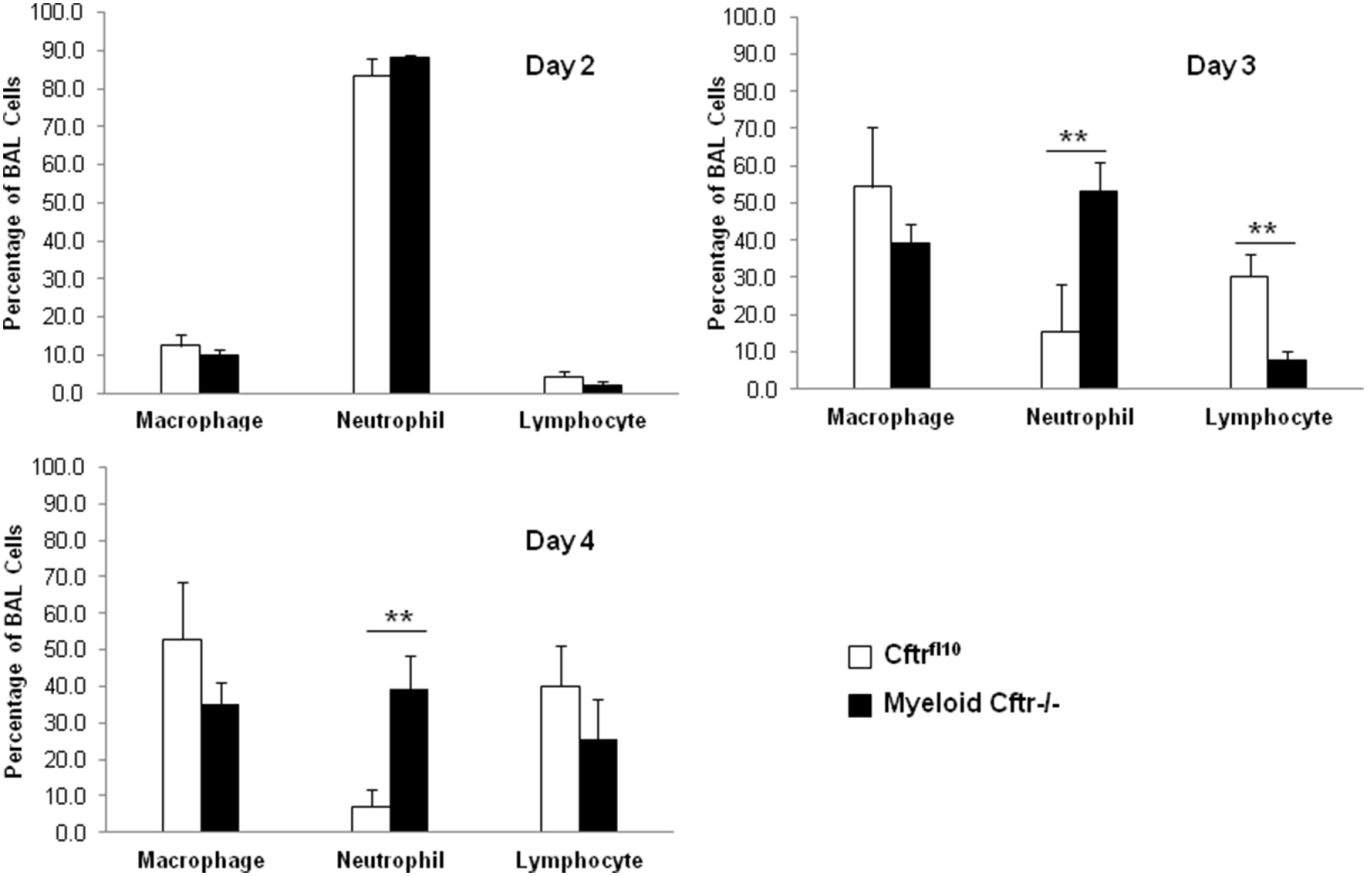
Abnormal profile of lung immune cells in Myeloid-Cftr−/− mice in response to lung PsA infection. Myeloid-Cftr−/− mice and Cftr^fl10^ control mice were intubated intratracheally with the agarose-embedded PsA at the sub-lethal dose (1×10^6^ cfu). BAL cells were collected and evaluated for differentials by cytospin and Giemsa staining. At Day 2 after PsA infection, neutrophils were the main constituent of the BAL cells (∼85%) in the lungs of both control mice and myeloid CF mice. In contrast, at Days 3 and 4 neutrophil percent in the Cftr^fl10^ control lungs reduced to ∼15% and ∼7%, respectively. However, the percentage of neutrophils in the Myeloid-Cftr−/− lungs dropped only to ∼52% at Day 3 and ∼39% at Day 4, which still remained dominant. Asterisks indicate statistically significant differences by Student’s t-test (n = 4, p<0.01).

We next asked whether the infected Myeloid Cftr−/− lungs recapitulate the characteristic neutrophilic inflammation in CF patients’ lungs. The control Cftr^fl10^ and Myeloid-Cftr−/− mice were intratracheally instilled with the sub-lethal dose of the agarose-embedded PsA (1×10^6^ cfu). At Days 2, 3 and 4, lung immune cells were harvested and differentials of these cells determined. Days 2–4 were chosen due to the high mortality rate during this time frame after lethal-dose PsA challenge, as shown in **Fig. 5**. Dynamics of neutrophils, macrophages and lymphocytes in the control and myeloid Cftr−/− lungs are displayed (**Fig. 7**). At Day 2, neutrophils were the predominant cells in all the lungs, occupying more than 80% of the total lung-recovered immune cells. There was no significant difference between the control and myeloid CF mice with regard to the percentage of each examined cell type. However, at Days 3 and 4 the control Cftr^fl10^ lungs exhibited receding of neutrophils and rising of macrophages and lymphocytes which became the major cell types. This represents a normal transition from early to late immunity, which is a typical process of resolution of infection and inflammation. Strikingly, the Myeloid-Cftr−/− lungs had a persistent neutrophilic inflammation and remained at the acute stage of immunity. This observation is consistent with the symptom of human CF lung disease. The data suggest that the myeloid CF mice have an intrinsic defect in neutrophil-mediated innate immunity, which contributes to the incomplete resolution of lung infection and infection.

## Discussion

CF lung disease is currently recognized as an epithelial and mucosal disease [49], in which immune cells are not considered as a primary player. Decades of research, centered on airway epithelia, has identified that airway CFTR dysfunction impairs transepithelial chloride and bicarbonate transport, which affects airway host defense. However, the epithelial defect alone cannot completely explain why CF lungs are predominantly infected by the opportunistic PsA, *Staphylococcus aureus* and *Haemophilus influenzae*, as opposed to the more virulent *Streptococcus pneumoniae* and *Klebsiella pneumoniae*, the common pathogens causing community-acquired pneumonias. We propose that the pathogen selection process involves host immune mechanisms.

CF lung disease must be triggered by lung infection, as fetal and newborn lungs of CF are functionally normal [50]. Thus, it is possible that CF lungs with defective host defense fail to eradicate the early invading microbes, leading to the development of chronic lung infection. Infected lungs are populated by not only the resident epithelial cells and tissue macrophages but also the mobilized immune cells. It is well established that the lung-recruited immune cells, largely neutrophils and monocytes, are an essential component in defending against extracellular bacterial infection [51]. The importance of this host defense mechanism is clearly exhibited by frequent or severe infection in patients with poor quantity or quality of circulating neutrophils such as in bone marrow failure [52], radio- or chemo-therapy [53], or inherited disorders of PMNs [54], [55]. Using the myeloid Cftr-inactivated mice, we have identified that the CFTR with Exon-10 deletion is expressed in neutrophils, but fails to target to the phagosomes. This engineered mutation closely mimics the common genetic ΔF508 mutation by affecting CFTR phagosomal targeting [31]. Myeloid-Cftr−/− mice have impaired neutrophil intraphagosomal HOCl production and compromised neutrophil microbial killing. These data thus validate our previous finding from CF patient neutrophils, demonstrating that CFTR chloride channel defect leads to microbicidal defect in neutrophils [30], [31], [32], [33].

The bacterial challenge and animal survival data (**Fig. 5**) have revealed that the lung-infected Myeloid-Cftr−/− mice have ∼30% higher mortality than control Cftr^fl10^ mice, suggesting that myeloid CFTR dysfunction alone has significant impact on lung host defense. More importantly, this study has identified a striking neutrophil aberrance in the Myeloid-Cftr−/− lungs. As shown in **Fig. 7**, the early stage of anti-infection immunity (Day 2) appears normal in Myeloid-Cftr−/− mice, as they can recruit a comparable number of neutrophils to the lungs as the control mice. However, at Day 3 and Day 4, neutrophil-mediated innate immunity fades away in the control lungs and is replaced by late immunity exemplified by predominant macrophages and lymphocytes. However, the Myeloid-Cftr−/− lungs fail to achieve such a transition. This is perhaps due to the incompetency of the neutrophil-mediated innate immunity which holds the lungs to a prolonged early immunity state. This postulation is supported by the long-observed pathology in CF lungs characterized by persistent neutrophilic inflammation.

In Myeloid-Cftr−/− mice, monocytes/macrophages also lack functional CFTR (**Fig. 1B**, and [35]). Due to the focus of this study, we did not assess the function of the CF macrophages. It is possible that the macrophage defect may also contribute to the overall lung host defense defect. Previous studies have reported that CF lung macrophages have impairment in bacterial killing [56], [57], [58] and exaggerated inflammatory response [4], [59]. However, macrophages are more specialized in antigen presentation and immune modulation than in direct bacterial killing. The reason is that this type of phagocyte lacks the complete assortment of antimicrobial agents as in neutrophils and has very limited expression of MPO. Consequently, macrophages produce significantly less HOCl [60]. The exact role of macrophages in direct lung clearance of Pseudomonas in the myeloid CF mice awaits further characterization.

Neutrophils heavily invest in the MPO-halide-H_2_O_2_ host defense system, discovered by Klebanoff and colleagues about half a century ago [22], [26], by producing MPO to the magnitude of ∼5% of the total cellular proteins. Because of redundant and overlapping activities of multiple arms of host defense mechanisms, the MPO-halide-H_2_O_2_ system is not always pronouncedly manifested unless the pathogens overwhelms the capacity of the other host defense mechanisms [28]. According to the HOCl biosynthesis (H_2_O_2_+H^+^+Cl^−^MPO→HOCl+H_2_O), there are four factors potentially limiting this reaction: H_2_O_2_, H^+^, Cl^−^ and MPO. MPO-deficient neutrophils exhibit a profound defect in Candida clearance and bacterial killing [61], [62], [63], [64], [65], [66]. Our data here provide the first evidence indicating that limited attainability of chloride, one of the substrates, diminishes the HOCl production and neutrophil microbial killing, which consequently spoils the phagocytic host defense. This finding establishes a novel link between a chloride channelopathy and a host defense failure, reiterating the vital role of the MPO-halide-H_2_O_2_ system.

In summary, our current study using the myeloid tissue-specific Cftr-inactivation mice clearly demonstrates the significant role of CFTR in neutrophil-mediated innate immunity. CFTR dysfunction in myeloid cell lineages alone significantly affects the lung host defense. Thus, CF lung disease should be defined by the defects of multiple types of cells, including lung-recruited immune cells.
